# The Pattern of Superficial Body Temperatures in Leisure Horses Lunged with Commonly Used Lunging Aids

**DOI:** 10.3390/ani9121095

**Published:** 2019-12-07

**Authors:** Malgorzata Maśko, Lukasz Zdrojkowski, Malgorzata Domino, Tomasz Jasinski, Zdzislaw Gajewski

**Affiliations:** 1 Department of Animal Breeding, Faculty of Animal Science, Warsaw University of Life Sciences (WULS–SGGW), 02-787 Warsaw, Poland; 2 Department of Large Animal Diseases with Clinic, Veterinary Research Centre and Center for Biomedical Research, Faculty of Veterinary Medicine, Warsaw University of Life Sciences (WULS–SGGW), 02-787 Warsaw, Poland; lukasz_zdrojkowski@sggw.pl (L.Z.); malgorzata_domino@wp.pl (M.D.); tomasz_jasinski@sggw.pl (T.J.); zdzislaw_gajewski@sggw.pl (Z.G.)

**Keywords:** rubber band, chambon, triangle side reins, lunging, head and neck position, leisure horses, thermography

## Abstract

**Simple Summary:**

In the training of horses, special lunging aids may be used to regulate head and neck position during exercise without the intervention of a rider. The rubber band and triangle side reins and the chambon have an impact on thoracolumbar kinematics and the motion of the fore- and hindlimbs. Lunging aids are assumed to modulate the work of horses’ muscles, which results in altering the superficial thermographic patterns. Thermography is a non-invasive, contactless imaging technique based on detecting emitted infrared radiation representing the temperature of the body surface, influenced by muscle metabolism and blood circulation. Training sessions for 16 horses performed in the study included exercises at walk, freely active trot, and canter. Surface temperatures of 11 regions of interest were evaluated on all images, corresponding to areas influenced by neck fixation and engaging hindquarters. In conclusion, thermography was shown as a useful tool in lunging aids’ usefulness evaluation. Different types of lunging aids influence the mobility of horse neck and back and its choice for leisure horses lunging should be made individually. Lunging aids change the surface temperature of different body parts during the leisure horse work on the lunge.

**Abstract:**

Background: The natural head and neck position (HNP) of horses differs from the position in horse riding when bit is used. The special lunging aids (LAs) are applied in order to modify HNP. Different types of LAs have the potential to affect the work of horse muscles and the superficial thermographic patterns (STPs). The effects of thre LAs on STPs of neck, chest, back, and hindquarters were investigated. Methods: Sixteen leisure horses were lunged with freely moving head (FMH), rubber band (RB), chambon (CH), and triangle side reins (TRs). The thermographic images (n = 896) were analyzed before/after lunging for mean temperatures (T_mean_) and minimum–maximum difference (T_diff_). Results: Superficial T_mean_ increased (*p* < 0.001) in cranial part of neck, back, thoracic area, and limbs after lunging regardless of LAs application or its type. In comparison to other LAs: With RB, T_mean_ was higher in regions of interest (ROIs) 2,7 and lower in ROIs 3–4 (*p* < 0.05); with CH, T_mean_ was higher in ROIs 2–4 and 7 (*p* < 0.01); and with TRs, T_mean_ was higher in ROIs 2–4,7,9–11 (*p* < 0.01). In ROIs 2–4 and 7, T_diff_ was lower with LAs than with FMH (*p* < 0.01) and in ROIs 9–10 with TRs. Conclusions: The choice of LAs should be dictated by the expected effect; however, all LAs increase the quality of the leisure horse lunging. LA use is more desirable than lunging with FMH.

## 1. Introduction

The natural head and neck position (HNP) is typical for horses without any load. The freely running horse lowers the head/neck base and relaxes the muscles with open head/neck angle (HNA) [1], whereas in saddle use, HNA often remains closed [2]. In the training of horses, special lunging aids (LAs) are applied in order to regulate HNP during exercise without the intervention of a rider [3,4]. The LAs modify HNP by forces acting on the bit, head, breast, girth, and withers. The rubber band (RB) and triangle side reins (TRs) close HNP, whereas the chambon (CH) opens it [5]. The impact of HNP on thoracolumbar kinematics [6] and the motion of the fore- and hindlimbs [5] was previously described; therefore, the LA may also be assumed to modulate the work of horses’ muscles and deep stabilizing structures of ligaments [7]. This modulation depends both on the differing degree of reins’ tension/elasticity and the length of the aids causing the more loose or tight attachment [5,7]. The RB represents compliant elastic reins, whereas TRs are the stiffest [8]. Every LA can be used with different length; therefore, the comparison between their types should consider the aids length individually matched to the horse or target HNP. 

The research was conducted on the leisure horses. Horses in leisure usage, which is recreation, differ sports usage, considered as competitive in Olympic disciplines on different levels, and racing usage, considered as participating in races [9,10]. The difference between sports or racing usage and leisure usage may be defined based on determining the activity initiator and the purposes of training. In sports or racing usage, only the human participants are responsible for taking the initiative during the training, which is conduct for specific purposes such as raising the skills needed in Olympic disciplines or races. Whereas, in a case of leisure usage, especially riding outdoors in the fields or the forest, also the horse may take the initiative under human control, and the main goal of the training is relaxation and active rest [11].

We suppose that an application of a different type of LAs has the potential to affect the work of horse muscles, which results in altering the superficial thermographic patterns (STPs), compared to lunging with a freely moving head (FMH). Differences between the freely moving and fixed head may affect blood circulation in the muscles because of the forced change of HNP [4]. The increase in blood circulation occurs when muscle tension in a particular region is increased [12], whereas insufficient blood circulation decreases muscle oxygenation and affects metabolism [13]. Superficial muscles blood circulation affects STP, which may be assessed with thermography. Thermography is a non-invasive, contactless imaging technique based on detecting emitted infrared radiation representing the temperature of body surface [14], influenced by muscle metabolism and blood circulation [15,16]. Soroko et al. (2012) described changes of back temperature distribution in response to training cycle [17], whereas Simon et al. (2006) presented temperatures of fore- and hindlimbs during training sessions on the treadmill [15]. The STP of the head/neck region was also assessed to determine differences in blood circulation in the musculature between hyperflexion and FMH [4]. In all these studies the specific areas of body surface were chosen for statistical analysis of thermographic data to quantify the thermographic images. Those areas, named further as regions of interest (ROIs), represent the work of the large muscle groups significantly involved in horse mobility in the neck, chest, back, and hindquarters, following Becker-Birck et al. (2013), Soroko et al. (2012), and Simon et al. (2006), respectively [4,15,17].

The objective of this study was to evaluate STPs of the different regions of the horse—the neck, chest, back, and hindquarters—as well as compare patterns obtained from the same horses after lunging with and without LAs. Three types of LAs were verified: rubber band, chambon, and triangle side reins.

## 2. Materials and Methods

Sixteen horses participated in the study (7 mares, 9 geldings, mean age 12.7 ± 3.6 years) of three Polish warmblood breeds (56% Malopolska; 11% Wielkopolska; 33% Polish Halfbred horse) and four horse handlers from the Animal Sciences Students Ridden Association. All horses were housed in individual stalls with the same management in the Didactic Stable of Horse Breeding Division at WULS. The horses were fed three times a day with a dose of oats and hay personalized to each horse to maintain optimal, healthy condition without obesity, and had daily access to a sandy paddock no shorter than 6 h per day. All horses were in daily leisure use, considered as recreational riding 1 to 2 h a day, five days a week, also including lunging with FMH and LAs. The thermal imaging was performed following the international veterinary standards [18], and the health status and the mouth condition were inspected before the experiment [19]. Horses were clinically healthy, with no dental disorders or any signs of ulceration in mouth examination, and demonstrated a comparable condition and athletic ability.

**Figure 1:**
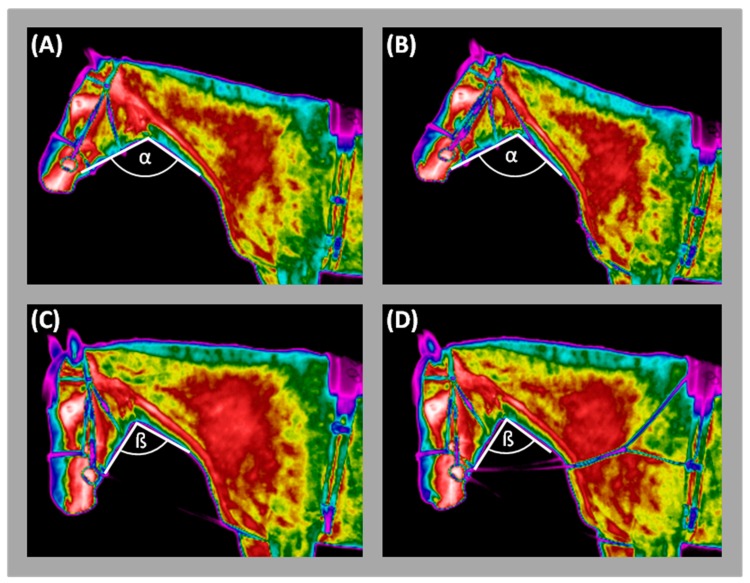
Thermographic image of horse lunged with: (**A**) freely moving head, (**B**) chambon, (**C**) rubber band, (**D**) triangle side reins. The open head and neck position marked with α: 110°–115°; the close head and neck position marked with ß: 85°–90°. The dashed lines marked the location of subsequent lunging aids.

All horses were tested using the same procedure in the same order: without aids, with RB, CH, and TRs. The LA was always used with comparable length adjusted to each horse basing on the HNA measured each time on the image. The HNA of a horse under these conditions was α: 110°–115° without reins and with CH (Figure 1A,B), while ß: 85°–90° with an RB or TRs (Figure 1C,D). The HNPs were tested for four consecutive days, one for each position.

A total of 896 images were taken before and after training sessions in an indoor ridden hall (ambient temperature 20.2 °C ± 1.1), protected from wind and sun radiation, and connected with the stable in which horses were housed. Images were taken using an infrared radiation camera (VIGOcam.v50, VIGOSystem S.A., Ozarow Mazowiecki, Poland) with an emissivity (e) that ranged from 0.99 to 1.00 and a temperature range 20–40 °C. The camera was handheld at a distance of approximately 1.2 m up from the horse, at right angles to the imaged plane—the lateral plane of the neck and hindquarters, the frontal plane of the chest, the dorsal plane of the back, and the caudal plane of hindquarters. 

Each data collection started with bringing one horse at a time from the stable, letting it stand in the middle of the riding hall to take the baseline pictures. Then training sessions started and included exercises in both directions at walk, freely active trot, and canter, individually adjusted to each horse for the appropriate tempo: walk (up to 1.5 m/s), trot (up to 4.0 m/s), and canter (up to 6.0 m/s). The total duration of each session was 50 min and included 10 min of walk, 10 min of trot, 5 min of canter, again 10 min of trot, again 5 min of canter, then 5 min of trot, and finally 5 min of walk. After the second imaging, the horses were walked on the rope to complete rest.

**Figure 2:**
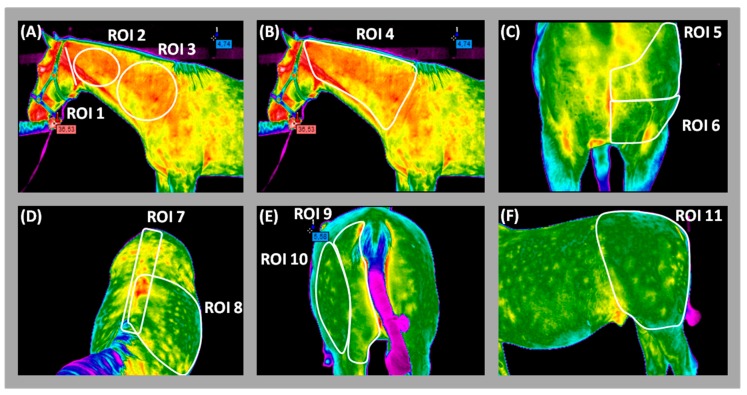
The regions of interest (ROIs) chosen for statistical analysis of thermographic data: (**A**) ROIs 1–3, (**B**) ROI 4, (**C**) ROIs 5–6, (**D**) ROIs 7–8, (**E**) ROIs 9–10, (**F**) ROI 11.

Surface temperatures of 11 regions of interest (ROIs 1–11) were evaluated on all 896 images according to criteria described in Table 1. Consecutive ROIs (1–4) correspond to areas influenced by neck fixation. Via analyses of ROIs 5–11, we are able to determine if and how fixation of neck obtained by lunging aids is altering muscles activity in other body parts of the horse. (Figure 2).

The symmetrical regions (ROIs 1–6, ROIs 8–11) were taken under consideration independently for left and right side. Each ROI was analyzed according to the protocol proposed by Becker-Birck et al. (2013) for the minimum (T_min_), maximum (T_max_), and mean (T_mean_) temperatures measured with the THERMCAM software (VIGOSystems S.A., Ozarow Mazowiecki, Poland). Then the minimum–maximum difference (T_diff_) was calculate based on T_min_ and T_max_ measures [4]. 

Obtained data were represented in the form of numerical series representing measurements before and after an effort, as well as among FMH and type of lunging aid used. Each data series was tested independently for univariate marginal distributions using a univariate Kolmogorov–Smirnov test. Most of the data series obtained for ROIs 1–11 in the examined HNPs (FMH, RB, CH, TRs) represented Gaussian distribution when the T_min_, T_max_, T_mean_, T_diff_ were taken under consideration. On the other hand, after effort, non-Gaussian distribution in the following data series was found: T_mean_ in ROIs 4, 6, 8 when the horses were lunged with FMH and RB; T_min_ in ROI 1 (CH) and ROI 7 (TRs); T_max_ in ROI 2 (RB) and ROI 6 (CH); as well as T_diff_ in ROI 6 (FMH) and ROI 8 (CH).

Firstly, data series of all measurements (T_min_, T_max_, T_mean_, T_diff_) obtained from the right and left side were compared using Unpaired t-test with Welch’s correction (for Gaussian distribution of all data series) or the Mann–Whitney test (for non-Gaussian distribution of at least one data series). Next, the data series of two measurements (T_mean_, T_diff_) were compared to estimate the differences between measurements among FMH and type of lunging aid used. For this purpose, one-way ANOVA test followed by Tukey’s multiple (for Gaussian distribution), or Kruskal–Wallis test with Dunn’s multiple comparisons test (for non-Gaussian distribution), was used. Finally, again the data series of two measurements (T_mean_, T_diff_) were tested using unpaired t-test with Welch’s correction (for Gaussian distribution) or the Mann–Whitney test (for non-Gaussian distribution) to distinguish the differences before and after an effort. All statistical analysis was performed using GraphPad Prism6 software (GraphPad Prism 6; GraphPad Software Inc., San Diego, CA, USA), where the significance level was established as *p* < 0.05.

## 3. Results

The values of T_min_, T_max_, T_mean_, and T_diff_ (data not shown) in all 11 analyzed ROIs did not show any differences between right and left side both before and after effort. T_mean_ was determined for each ROI, and the values of T_min_, T_max_ were used to calculate T_diff_; therefore, the mean ± SD values of T_mean_ and T_diff_ were summarized in Table 2 and Table 3, respectively, to demonstrate the changes in the pattern of superficial body temperatures in leisure horses lunged with commonly used lunging aids.

**Figure 3:**
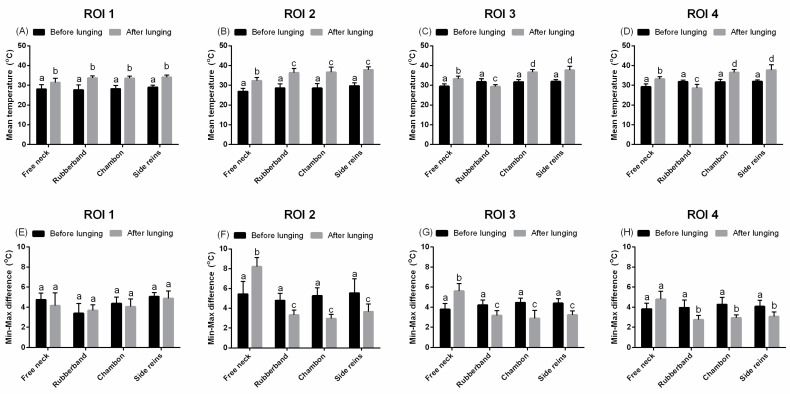
The superficial thermographic patterns of regions of interest (ROIs) of the neck. Horses lunged with: freely moving head, rubber band, chambon, and triangle side reins. (**A**,**E**) ROI 1, (**B**,**F**) ROI 2, (**C**,**G**) ROI 3, (**D**,**H**) ROI 4, (**A**–**D**) T_mean_, (**E**–**H**) T_diff_. Lower case letters indicate differences between measurements before and after effort, as well as differences among freely moving head and type of lunging aid used. The differences were significant for *p* < 0.05. All values were reported as mean + SD.

**Figure 4:**
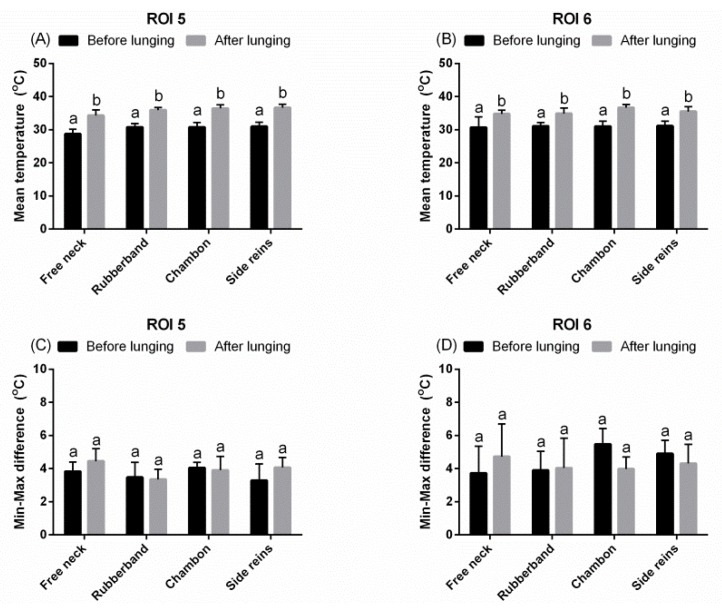
The superficial thermographic patterns of regions of interest (ROIs) of the chest. Horses lunged with: freely moving head, rubber band, chambon, and triangle side reins, before and after effort. (**A**,**C**) ROI 5, (**B**,**D**) ROI 6, (**A**,**B**) T_mean_, (**C**,**D**) T_diff_. Lower case letters indicate differences between measurements before and after an effort, as well as differences among freely moving head and type of lunging aid used. The differences were significant for *p* < 0.05. All values were reported as mean + SD.

**Figure 5:**
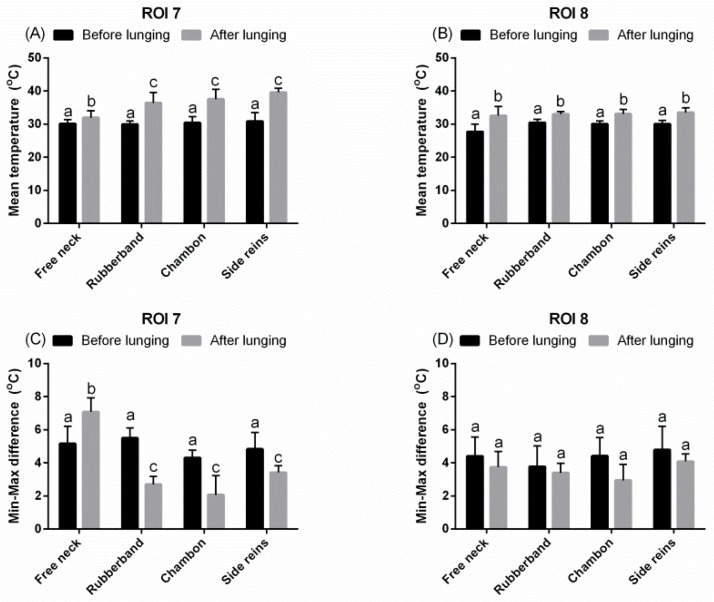
The superficial thermographic patterns of regions of interest (ROIs) of the back. Horses lunged with: freely moving head, rubber band, chambon, and triangle side reins, before and after effort. (**A**,**C**) ROI 7, (**B**,**D**) ROI 8, (**A**,**B**) T_mean_, (**C**,**D**) T_diff_. Lower case letters indicate differences between measurements before and after an effort, as well as differences among freely moving head and type of lunging aid used. The differences were significant for *p* < 0.05. All values were reported as mean + SD.

**Figure 6:**
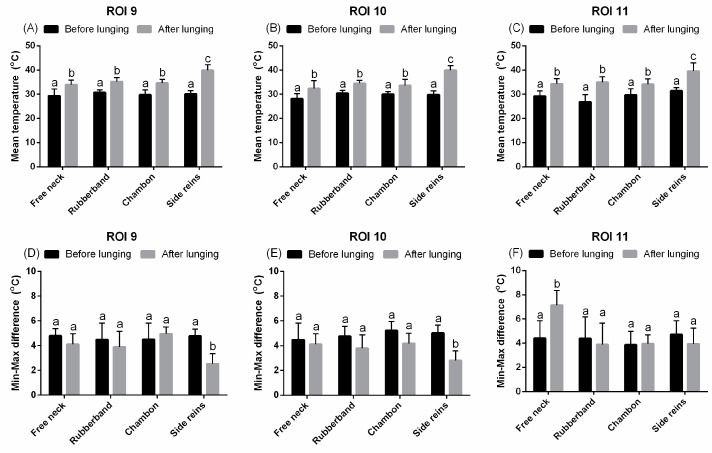
The superficial thermographic patterns of regions of interest (ROIs) of the hindquarters. Horses lunged with: freely moving head, rubber band, chambon, and triangle side reins, before and after effort. (**A**,**D**) ROI 9, (**B**,**E**) ROI 10, (**C**,**F**) ROI 11, (**A**,**B**) T_mean_, (**C**,**D**) T_diff_. Lower case letters indicate differences between measurements before and after an effort, as well as differences among freely moving head and type of lunging aid used. The differences were significant for *p* < 0.05. All values were reported as mean + SD.

In the majority of analyzed ROIs of the neck (ROIs 1–2), chest (ROIs 5–6), back (ROIs 7–8), and hindquarters (ROIs 9–11), the T_mean_ increased after lunging with both FMH and LAs (*p* < 0.001; Figure 3A–D, Figure 4A,B, Figure 5A,B, and Figure 6A–C).

In the neck regions, T_mean_ changed depending on the type of reins in comparison to FMH (Figure 3A–D). After an application of RB, T_mean_ was higher in ROI 2 and lower in ROI 3 and ROI 4 (*p* < 0.05; Figure 3B–D). After application of CH and TRs, T_mean_ was higher in ROIs 2–4 (*p* < 0.01; Figure 3B–D). In ROIs 2–4, T_diff_ was lower in comparison to FMH, regardless of the type of LAs applied (*p* < 0.01; Figure 3E–H). In ROI 1, no differences were observed between fixed and extended HNP for both T_mean_ and T_diff_ (Figure 3A,E).

In ROI 7, T_mean_ was higher and T_diff_ was lower when horses were lunged with LAs than with FMH, regardless of their type (*p* < 0.01; Figure 5A,C). However, no differences were observed in the ROI 8 both for T_mean_ and T_diff_ (Figure 5B,D).

**Figure 7:**
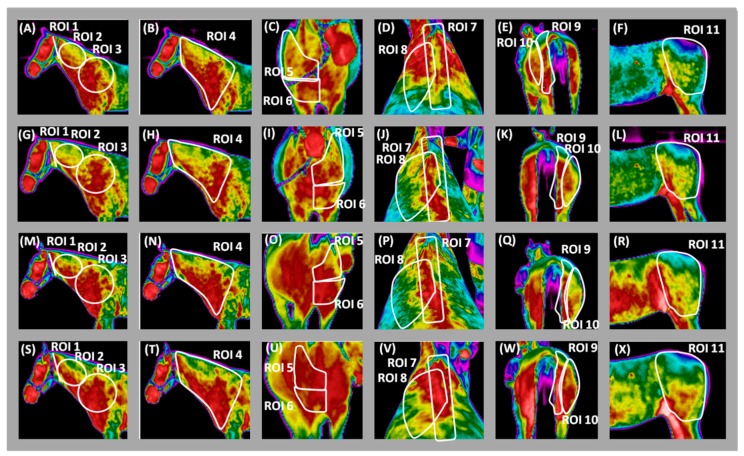
The thermographic images after training session with freely moving head (**A**–**F**), rubber band (**G**–**L**), chambon (**M**–**R**), and triangle side reins (**S**–**X**) of the same horse with the marked regions of interest (ROIs) chosen for statistical analysis: (**A**,**G**,**M**,**S**) ROIs 1–3; (**B**,**H**,**N**,**T**) ROI 4; (**C**,**I**,**O**,**U**) ROIs 5–6; (**D**,**J**,**P**,**V**) ROIs 7–8; (**E**,**K**,**Q**,**W**) ROIs 9–10; (**F**,**L**,**R**,**X**) ROI 11.

The values of T_mean_ were higher in all three regions of hindquarters (ROIs 9–11) and the T_diff_ was lower in ROIs 9–10 when horses were lunged with TRs, compared to the other experimental groups (*p* < 0.0001; Figure 6A–F). Regarding ROI 11, T_diff_ only increased when horses were lunged without aids (*p* < 0.05; Figure 6F). No other differences were found for any of the parameters and regions analyzed depending on the type of the examined LA (Figure 3E, Figure 4C,D and Figure 5D). Example of pictures depicting thermographic patterns after training session in FMH and with LA is presented in Figure 7.

## 4. Discussion

Leisure horses lunging with all LAs caused an increase in the STP in specific ROIs. These changes seem to be related to the magnitude of physical activity, what stays in line with previous studies on exercised horses [4,20,21]. Vertical displacement of anatomic landmarks, such as head, neck, withers, and sacrum, in the sagittal plane, is asymmetric during trotting in a circle, and if it is too intensive or lasts too long, may lead to the overload of the muscles on each side [22] and may lead to the increase of the STP [15,16]. The effect of asymmetric forces acting on the horse during lunging may be reduced when the individual horse had developed the mechanisms to deal with it [22]. The mechanisms could slightly differ between horses [22]; however, the possibility of their actions may indicate the welfare of a horses’ work on the circle [4,5]. Therefore, the lack of differences between right and left side indicated a correctly selected research methodology, a well-chosen research group (symmetrical patterns before lunging), as well as even work on the lunge (symmetrical patterns after lunging). Moreover, the asymmetric heat distribution is an indicator of malfunctioning horses’ mobility in the region of the neck [23], chest [15], back [14], and hindquarters [23]. Mean STP increased as the horses were lunged regardless of LA; however, rein type affected the temperature in different ROIs. 

The influence of head fixation on the STP in the neck region was previously discussed, with higher T_diff_ only in the cranial region of the neck when horses were lunged with a hyperflexed HNP in comparison to FMH [4]. An increase in both T_mean_ and T_diff_ was noticed when the use of TRs (restriction on head/neck movements), CH (stiffly limiting the rise of the head), and RB (flexibly limiting the rise of the head) changed the STP of the cranial region of the neck (ROI 2) significantly more than FMH. The head position was stabilized by LAs [24]; therefore, an increase in the blood circulation in the musculature of the cranial region of the neck is understandable. The stabilization of the head position on the bit was considered as a soft restriction of head movement associated with the highest comfort with the open HNA for CH (α: 110–115) or the close HNA for RB and TRs (ß: 85–90°), which did not cause pain in those angles. We also found no differences in the line from the guttural angle to nape (ROI 1) in T_mean_ and T_diff_, but changes depending on the rein type were found in the caudal part of the neck (ROI 3). 

When the neck descends in relation to the trunk, head position is controlled by tension in the elastic nuchal ligament (passively) and by tension of musculature (actively) [25,26]. Horses may reduce the active muscular input, from 55% to 31% of work, by taking advantage of the elastic components energy storage capabilities. In FMH, the nuchal ligament is responsible for all passive work and involved 33% of work in HNP stabilization [26], whereas LAs may play a role in energy conservation during movement in a manner similar to the nuchal ligament [7]. Our data suggested that the type of LA may affect the capability of elastic energy stored, similarly to the nuchal ligament [26]. When the head/neck descends, LAs are stretched proportionally to their elasticity, then release the energy like a spring when the head/neck raises. We found the lowest T_mean_ in ROIs 3–4 when horses were lunged with a compliant elastic RB in opposition to the increase in ROIs 3 and 4 with CH and TRs (Figure 2 and Figure 3). Based on the increase of T_mean_ in those ROIs, it may be concluded that TRs restricted the action of passive anatomical components and promoted the action of epaxial muscles, which may lead to their strengthening [27]. The nuchal ligament is prolonged caudally into the supraspinous ligament, responsible for passive work of the back [26]. Thereby the active muscular input of the back increases, what is visible as an increase of T_mean_ in ROI 7 but not in ROI 8, regardless of the LA type. In the previous research, the influence of HNP on the kinematics of the back of ridden horses indicated that restricting movement of the head/neck alters the movement of the back and stride characteristics [3]. High HNP reduces stride length and flexion–extension movement of the caudal back [3]. In our experiment HNP was fixed and lowered, which contributed for an effect on back superficial temperature pattern. The use of the TRs seems to promote not only the action of epaxial muscles, indicated by T_diff_ increasing in ROI 7, but may provide a transfer of force between the hindlimbs and the back, indicated by T_mean_ increasing in ROIs 9-11 and T_diff_ decreasing in ROIs 9–10. Leisure horses often try to compensate insufficient back movement by increasing retraction of the forelimbs [28]. Therefore training exercises should activate the mobility of the back, hence engage hindlimbs motion. The highest engagement of hindlimbs was represented by the highest physical activity of the muscles of the hindquarters, since it was reflected by the highest T_mean_ in ROIs 9–11 and the lowest T_diff_ in ROIs 9–10 when horses were lunged with TRs.

Lunging with aids has a positive effect on the variability of T_mean_ and T_diff_ in ROIs 2–3, 7, 9–10, whereas lunging in hyperflexion was associated with a less homogenous pattern [4]. When horses were lunged with LAs, it allowed a lower variation of temperature in ROIs 2–4 and ROI 7 in comparison to the pattern before lunging or lunging with FMH. Lunging with TRs was even more beneficial because it additionally changed in thermographic pattern in ROIs 9–10. Lunging without aids was associated with a less variation in STPs in the described regions. Therefore, it may be concluded that horses’ work on the lunge with LAs is more beneficial than lunging with a FMH of those horses’ daily leisure work. Lunging without aids was associated with less variations in the STPs in described regions; therefore, it may be concluded that horses’ work on the lunge with LAs is more beneficial than lunging with an FMH. The proper use of LAs activates the axial musculature and raises back mobility [6].

The changes in superficial temperatures may be caused by a combination of physical activity and physiological stress responses [15,29,30], which is not easy to differentiate. Interestingly, infrared thermography has been used to identify physiological stress in horses based on the evaluation of eye temperature [31], which showed positive correlations with heart rate, heart rate variability, stress related behaviors, and cortisol concentration [32]. However, Becker-Birck et al. (2013) concluded that a transient and moderate hyperflexion in horses lunged at moderate speed and not touched with the whip did not elicit a pronounced stress response in horses. They also did not observe overt aversive or stress related behavior of the horse [4]. In the present study, hyperflexion was not used, but instead a much more neutral and stressless HNP with the open HNA (α: 110°–115°) and the close HNA (ß: 85°–90°), especially since lunging with LA was not a new element of those horses’ daily leisure work. The other conditions of this experiment were comparable; therefore, we can make a cautious assumption that the impact of stress on our results was insignificant. However, in our study the salivary or blood cortisol concentrations, heart rate, heart rate variability, and stress behaviors were not examined. Further investigation regarding the evaluation of eye temperature should be considered.

One of the practical applications of our results relates to the selection of LAs. In our opinion, LAs should be chosen depending on the purpose of training exercises, especially in the leisure type of work. When a leisure horse is working with a beginner rider, the use of LA such as RB is recommended in order to substitute the passive work of equines’ neck and back. We support the statement of Clayton et al. (2012) that the elastic component is likely to be a beneficial addition in terms of reducing maximal tension and loading rate [7]. However, when a horse is working on the lunge, TRs seems to be a better option for the improvement of the forward extension of hind limbs and perhaps also the horses’ riding quality. After their application, the largest superficial temperature variations were visible for ROIs 3, 4, 7, and, interestingly, ROIs 9–11. Exercises, also understood as lunge work, that target the cervical region may not only have a mobilizing effect on the cervical and thoracolumbar spine, but also activate and strengthen the epaxial and hypaxial muscles throughout the cervical, thoracic, and lumbar regions and thus may alter functional movement patterns [8].

## 5. Conclusions

Thermography was shown as a useful tool in determining changes of STP in horses after lunging, both on LAs and with FMH. Application of different types of LAs changes the STP, and probably also the mobility of horses’ neck and back. The choice of LA used in leisure horses lunging should be made individually. Because a small research group was used, we only suppose that the RB may decrease muscle activity of equine neck and back, whereas TRs can play a role in activation of neck, back, and hindquarters mobility. However, regardless of the choice made, LAs improve the thermographic pattern of the leisure horses during work on the lunge, and their proper use is probably more desirable than lunging with FMH.

## Figures and Tables

**Table 1:** The characteristic of regions of interest (ROIs) taken into consideration in the examined superficial thermographic patterns.

No	Shape of ROI	Range of ROI
ROI 1	a line	from the highest point of the horses’ nape of the neck to the ventral guttural angle
ROI 2	an ellipse	from the caudal aspect of the mandible to the ventral and dorsal lines of the neck
ROI 3	a circle	from the cranial brim of the saddle flap onto the neck and from the height of the shoulder joint to the upper aspect of the mane
ROI 4	an irregular, trapezoidal	from the line between the highest point of the neck and the ventral guttural angle to the line between the height of the shoulder joint and the highest spinous process of thoracic vertebrae, represents whole neck muscles work
ROI 5	an irregular, L-shaped	from the upper half of the middle chest line, horizontally to and along the lateral, upper edge of the pectoral muscles up to the middle of the spine of scapula
ROI 6	an irregular, trapezoidal	from the lower half of the middle chest line, horizontally to and along the lateral, lower edge of the pectoral muscles down to the manubrium of the sternum
ROI 7	a rectangle	from the highest spinous process of thoracic vertebrae to the last sacral vertebrae
ROI 8	an irregular, barrel-shaped	from the middle of thoracic vertebrae along the last rib to the widest line of the chest and up to the highest spinous process of thoracic vertebrae along the caudal edge of the scapula
ROI 9	an irregular, hourglass-shaped	from the highest point of ilium down to the knee joint along the tail contour to the lateral edge of the semitendinosus muscle
ROI 10	an irregular, fusiform	from anterior superior iliac spine to the knee joint, between the lateral edge of semitendinosus muscle and the lateral edge of the hindquarters
ROI 11	an irregular, barrel-shaped	from sacral vertebrae down to the knee joint along the caudal edge of hindquarters and along patella up to the coxal tuber

**Table 2:** The values (mean ± SD) of T_mean_ obtained before and after lunging with: freely moving head, rubber band, chambon, and triangle side reins. The values represent the superficial thermographic patterns of regions of interest (ROIs) of the neck (ROIs 1–2), chest (ROIs 5–6), back (ROIs 7–8), and hindquarters (ROIs 9–11). The first superscript letters (^a,b^) indicate differences between measurements before and after an effort independently for each head and neck position. The second superscript letters (^v,x,y,z^) indicate differences between freely moving head and type of lunging aid used before (^v^) and after (^x,y,z^) effort. The differences were significant for *p* < 0.05, independently for each row.

Lunging	Freely Moving Head		Rubber Band		Chambon		Triangle Side Reins	
ROIs	Before Lunging	After Lunging	Before Lunging	After Lunging	Before Lunging	After Lunging	Before Lunging	After Lunging
**ROI 1**	28.05 ± 2.24 ^a,v^	31.36 ± 2.18 ^b,x^	27.67 ± 2.52 ^a,v^	33.67 ± 1.15 ^b,x^	28.17 ± 1.76 ^a,v^	33.60 ± 0.96 ^b,x^	29.00 ± 1.00 ^a,v^	34.11 ± 1.00 ^b,x^
**ROI 2**	26.91 ± 1.53 ^a,v^	32.31 ± 1.67 ^b,x^	28.64 ± 2.03 ^a,v^	36.37 ± 2.15 ^b,y^	28.55 ± 2.36 ^a,v^	36.64 ± 2.60 ^b,y^	29.72 ± 1.60 ^a,v^	37.87 ± 1.42 ^b,y^
**ROI 3**	29.51 ± 1.19 ^a,v^	33.29 ± 1.37 ^b,x^	31.77 ± 1.60 ^a,v^	29.38 ± 0.95 ^b,y^	31.68 ± 1.25 ^a,v^	36.74 ± 1.34 ^b,z^	31.96 ± 0.93 ^a,v^	37.68 ± 1.95 ^b,z^
**ROI 4**	29.21 ± 1.59 ^a,v^	33.09 ± 1.36 ^b,x^	31.77 ± 0.88 ^a,v^	28.62 ± 1.83 ^b,y^	31.73 ± 1.30 ^a,v^	36.57 ± 1.42 ^b,z^	31.98 ± 0.83 ^a,v^	37.77 ± 2.26 ^b,z^
**ROI 5**	28.73 ± 1.47 ^a,v^	34.33 ± 1.69 ^b,x^	30.77 ± 1.13 ^a,v^	35.92 ± 0.78 ^b,x^	30.78 ± 1.39 ^a,v^	36.43 ± 1.07 ^b,x^	30.98 ± 1.26 ^a,v^	36.66 ± 1.02 ^b,x^
**ROI 6**	30.69 ± 3.17 ^a,v^	34.83 ± 1.09 ^b,x^	31.10 ± 1.04 ^a,v^	34.89 ± 1.68 ^b,x^	31.02 ± 1.54 ^a,v^	36.64 ± 1.02 ^b,x^	31.15 ± 1.44 ^a,v^	35.52 ± 1.41 ^b,x^
**ROI 7**	30.15 ± 1.20 ^a,v^	32.00 ± 2.01 ^b,x^	29.99 ± 1.00 ^a,v^	36.44 ± 3.13 ^b,y^	30.45 ± 1.85 ^a,v^	37.57 ± 3.01 ^b,y^	30.85 ± 2.69 ^a,v^	39.61 ± 1.25 ^b,y^
**ROI 8**	27.72 ± 2.27 ^a,v^	32.55 ± 2.77 ^b,x^	30.46 ± 0.96 ^a,v^	33.00 ± 0.75 ^b,x^	30.08 ± 0.89 ^a,v^	33.12 ± 1.38 ^b,x^	30.05 ± 1.11 ^a,v^	33.53 ± 1.39 ^b,x^
**ROI 9**	29.36 ± 2.78 ^a,v^	33.90 ± 1.90 ^b,x^	30.69 ± 1.09 ^a,v^	35.16 ± 1.61 ^b,x^	29.77 ± 1.97 ^a,v^	34.66 ± 1.50 ^b,x^	30.14 ± 1.28 ^a,v^	39.89 ± 2.30 ^b,y^
**ROI 10**	28.13 ± 2.13 ^a,v^	32.35 ± 3.29 ^b,x^	30.44 ± 1.11 ^a,v^	34.50 ± 1.20 ^b,x^	30.03 ± 1.08 ^a,v^	33.58 ± 2.53 ^b,x^	29.84 ± 1.53 ^a,v^	39.93 ± 1.84 ^b,y^
**ROI 11**	29.12 ± 2.22 ^a,v^	34.27 ± 2.12 ^b,x^	26.83 ± 3.01 ^a,v^	34.94 ± 2.26 ^b,x^	29.72 ± 2.51 ^a,v^	34.06 ± 2.21 ^b,x^	31.36 ± 1.37 ^a,v^	39.56 ± 3.40 ^b,y^

**Table 3:** The values (mean ± SD) of T_diff_ obtained before and after lunging with: freely moving head, rubber band, chambon, and triangle side reins. The values represent the superficial thermographic patterns of regions of interest (ROIs) of the neck (ROIs 1–2), chest (ROIs 5–6), back (ROIs 7–8), and hindquarters (ROIs 9–11). The first superscript letters (^a,b^) indicate differences between measurements before and after an effort independently for each head and neck position. The second superscript letters (^v,x,y^) indicate differences between freely moving head and type of lunging aid used before (^v^) and after (^x,y^) effort. The differences were significant for *p* < 0.05, independently for each row.

Lunging	Freely Moving Head		Chambon		Rubber Band		Triangle Side Reins	
ROIs	Before Lunging	After Lunging	Before Lunging	After Lunging	Before Lunging	After Lunging	Before Lunging	After lunging
**ROI 1**	4.74 ± 0.67 ^a,v^	4.17 ± 1.26 ^a,x^	3.40 ± 0.98 ^a,v^	3.69 ± 0.55 ^a,x^	4.36 ± 0.6 5 ^a,v^	4.05 ± 0.78 ^a,x^	5.05 ± 0.41 ^a,v^	4.89 ± 0.73 ^a,x^
**ROI 2**	5.44 ± 1.29 ^a,v^	8.22 ± 0.93 ^b,x^	4.78 ± 0.72 ^a,v^	3.32 ± 0.50 ^b,y^	5.27 ± 0.79 ^a,v^	2.95 ± 0.43 ^b,y^	5.55 ± 1.43 ^a,v^	3.65 ± 0.78 ^b,y^
**ROI 3**	3.79 ± 0.57 ^a,v^	5.61 ± 0.74 ^b,x^	4.20 ± 0.53 ^a,v^	3.17 ± 0.48 ^b,y^	4.46 ± 0.45 ^a,v^	2.90 ± 0.79 ^b,y^	4.39 ± 0.46 ^a,v^	3.24 ± 0.40 ^b,y^
**ROI 4**	3.83 ± 0.57 ^a,v^	4.79 ± 0.81^a,x^	3.95 ± 0.78 ^a,v^	2.76 ± 0.41 ^b,y^	4.27 ± 0.70 ^a,v^	2.93 ± 0.31 ^b,y^	4.07 ± 0.61 ^a,v^	3.08 ± 0.46 ^b,y^
**ROI 5**	3.81 ± 0.56 ^a,v^	4.46 ± 0.75 ^a,x^	3.48 ± 0.91 ^a,v^	3.36 ± 0.61 ^a,x^	4.05 ± 0.33 ^a,v^	3.91 ± 0.82 ^a,x^	3.30 ± 0.98 ^a,v^	4.07 ± 0.61 ^a,x^
**ROI 6**	3.73 ± 1.63 ^a,v^	4.73 ± 1.97 ^a,x^	3.90 ± 1.14 ^a,v^	4.04 ± 1.80 ^a,x^	5.48 ± 0.95 ^a,v^	3.98 ± 0.73 ^a,x^	4.90 ± 0.81 ^a,v^	4.32 ± 1.15 ^a,x^
**ROI 7**	5.17 ± 1.04 ^a,v^	7.08 ± 0.84 ^b,x^	5.50 ± 0.61 ^a,v^	2.70 ± 0.49 ^b,y^	4.31 ± 0.46 ^a,v^	2.07 ± 1.16 ^b,y^	4.84 ± 1.01 ^a,v^	3.42 ± 0.41 ^b,y^
**ROI 8**	4.40 ± 1.15 ^a,v^	3.74 ± 0.94 ^a,x^	3.77 ± 1.26 ^a,v^	3.40 ± 0.58 ^a,x^	4.42 ± 1.12 ^a,v^	2.93 ± 0.98 ^a,x^	4.79 ± 1.41 ^a,v^	4.08 ± 0.46 ^a,x^
**ROI 9**	4.79 ± 0.57 ^a,v^	4.12 ± 0.84 ^a,x^	4.48 ± 1.33 ^a,v^	3.88 ± 1.27 ^a,x^	4.50 ± 1.32 ^a,v^	4.96 ± 0.54 ^a,x^	4.78 ± 0.56 ^a,v^	2.53 ± 0.82 ^b,y^
**ROI 10**	4.48 ± 1.33 ^a,v^	4.12 ± 0.84 ^a,x^	4.76 ± 0.80 ^a,v^	3.78 ± 1.08 ^a,x^	5.22 ± 0.73 ^a,v^	4.91 ± 0.82 ^a,x^	5.04 ± 0.61 ^a,v^	2.81 ± 0.77 ^b,y^
**ROI 11**	4.40 ± 1.44 ^a,v^	7.13 ± 1.23 ^b,x^	4.41 ± 1.78 ^a,v^	3.90 ± 1.75 ^a,y^	3.87 ± 1.10 ^a,v^	3.96 ± 0.76 ^a,y^	4.71 ± 1.14 ^a,v^	3.94 ± 1.30 ^a,y^
